# Public Health Approaches and Barriers to Educating Providers about Hereditary Breast and Ovarian Cancer Syndrome

**DOI:** 10.3390/healthcare4010019

**Published:** 2016-03-11

**Authors:** Angela M. Trepanier, Laura Supplee, Lindsey Blakely, Jenna McLosky, Debra Duquette

**Affiliations:** 1 Center for Molecular Medicine and Genetics, Wayne State University, 540. E. Canfield, Detroit, MI 48154, USA; 2 Medical Genetics, Spectrum Health, 25 Michigan Street NE, Grand Rapids, MI 49503, USA; laura.supplee@spectrumhealth.org; 3 Saint Joseph Mercy Hospital, 5301 East Huron River, Ypsilanti, MI 48197, USA; lindsey.blakely@stjoeshealth.org; 4 InformedDNA, 360 Central Ave, St Petersburg, FL 33701, USA; jmclosky@informeddna.com; 5 Michigan Department of Health and Human Services, 201 Townsend, Lansing, MI 48909, USA; duquetted@michigan.gov

**Keywords:** hereditary breast and ovarian cancer syndrome (HBOC), public health initiative, genetic counseling, provider education

## Abstract

The Michigan Department of Health and Human Services implemented and evaluated two initiatives designed to enhance provider knowledge of patients appropriate for breast and/or ovarian cancer genetic risk assessment and hereditary breast and ovarian cancer (HBOC) syndrome testing. The first initiative targeted select providers who had diagnosed patients meeting HBOC risk criteria. Specifically, the initiative used 2008–2009 state cancer registry data to identify all providers who had diagnosed breast cancers in women ≤50 years of age, male breast cancers, and ovarian cancers in four health systems with newly established cancer genetics clinics. Using a method coined bidirectional reporting (BDR), reports highlighting how many of these cases each provider had seen were generated and mailed. Reports on 475 cancers (9.5% of the 5005 cases statewide meeting criteria) were sent to 69 providers with information about how and why to refer such patients for genetic counseling. Providers who received a report were contacted to assess whether the reports increased awareness or resulted in action (genetic counseling/referral). Based on the few responses received, despite multiple attempts to contact, and attrition rate, it is not possible to ascertain the impact of this initiative on providers. However the project resulted in the MDHHS identifying which providers see the largest proportion of at-risk patients, creating an opportunity to target those providers with HBOC education efforts. The second initiative involved creating and broadly disseminating an online, interactive case-based educational module to increase awareness and referral decisions for HBOC using high- and low-risk patient scenarios. A total of 1835 unique users accessed the module in a one year. Collectively the users viewed topic pages 2724 times and the interactive case studies 1369 times. Point of care tools (fact sheets) were viewed 1624 times and downloaded 764 times. Satisfaction among the subset of users applying for continuing medical education credit was high. The online educational module had a much broader reach than the bidirectional reporting initiative but to a self-selected audience. Combining targeted and broad-based provider education efforts may be a better way to increase HBOC awareness in the target audience, starting with those providers seeing the largest proportion of patients at risk.

## 1. Introduction

Healthy People 2020 includes a genomics objective that aims to “increase the proportion of women with a family history of breast and/or ovarian cancer who receive genetic counseling” [1]. An estimated 5%–10% of cases of breast cancers and 15% of invasive ovarian cancers are related to hereditary breast and ovarian cancer syndrome (HBOC) [2]. Guidelines for the clinical management of individuals with HBOC are available and are quite different from population screening guidelines for breast and ovarian cancer [3,4]. Preventive measures, including risk reducing bilateral salpingo-oophorectomy, have been shown to reduce cancer risk and all-cause mortality [5,6,7]. As such, appropriate identification and referral of individuals at risk for HBOC is necessary and could be life-saving [8].

The discovery that mutations in two genes, *BRCA1* and *BRCA2* [9,10], are associated with HBOC led to the availability of genetic testing to identify individuals at risk in the mid-1990s. In response, various organizations began to develop strategies for educating providers about how to identify at-risk individuals and referral guidelines for genetic counseling and testing [11,12].

Referral guidelines have evolved as more evidence has accrued regarding who most benefits from HBOC genetic counseling (GC) and genetic testing (GT). Based on a systematic evidence review [13], the United States Preventative Services Task Force (USPSTF) recommends that unaffected women at increased risk for harboring a *BRCA1/2* mutation have GC and then if indicated GT [14,15]. Conversely, USPSTF recommends against routine GC and GT for women whose family histories are not indicative of increased risk [14,15]. Based on the evidence, the United States Centers for Disease Control and Prevention’s Office of Public Health Genomics has classified the identification of individuals at risk for HBOC with referral for GC and GT as a Tier 1 genomics application [16]. Tier 1 applications are those that have “significant potential for positive impact on public health based on available evidence-based guidelines and recommendations.”

Guidelines regarding when to refer individuals already diagnosed with cancer for HBOC GC and GT are also available. Having a breast cancer diagnosis at or before age 50, ovarian cancer at any age, male breast cancer, triple negative breast cancer, two breast primaries, and/or a significant family history of breast or ovarian cancer are considered appropriate indications [3,17,18]. Despite the many referral guidelines available, provider knowledge of HBOC remains a barrier to appropriate identification and referral of patients at risk. Studies have shown that physicians lack basic knowledge concerning the genetics of HBOC, have difficulty recognizing individuals who may be at an increased risk [19], and/or do not know how to provide cancer GC services [20]. Inaccurate risk assessment can result in under-utilization of GC services as well as inappropriate referrals [21,22,23]. A systematic review identified an overall need to improve cancer genetics education, increase awareness of genetics services, and increase family history documentation among health care providers [24]. 

In order to address provider barriers and to increase provider knowledge and confidence regarding appropriate cases to refer for GC for HBOC, the Michigan Department of Health and Human Services (MDHSS) Cancer Genomics Program, through a 2011–2014 cooperative agreement with the Centers for Disease Control and Prevention (CDC), implemented two different case-based provider education initiatives. In Michigan, about 1 in 10 women have a significant family history of breast or ovarian cancer and should be considered for referral for genetic services [25]. However, approximately 90% of these women have not had GC [25]. The first initiative (2013) used existing data from the Michigan Cancer Surveillance Program (MCSP) to identify providers who had seen patients at risk of HBOC and highlight these cases (back) to the providers- a method coined “bidirectional reporting.” The second MDHHS provider education initiative utilized national and state partnerships to create a new online HBOC module approved for continuing medical education (CME) credit. The module used case scenarios to highlight key concepts regarding HBOC risk assessment, GC, GT, and referral.

The purpose of this manuscript is to describe the methods used to implement and evaluate these two initiatives and outcomes of the evaluations. Overall, the impact of the BDR project on increasing provider awareness of HBOC could not be evaluated; the online educational module reached a broad audience and was well-received by the subset that requested CME credit. However, access to the module was limited to those seeking out education about HBOC. Combining the approaches may be one way to increase awareness, and ultimately, appropriate identification of individuals at risk of HBOC, starting with the providers who diagnose the largest proportion of at risk patients.

## 2. Experimental Section

### 2.1. Bidirectional Reporting

In 2013, MDHHS implemented a bidirectional reporting initiative, targeting four specific health systems (centers) with new cancer genetic counseling clinics. Three of these clinics were in previously underserved areas (*i.e.*, nearest cancer genetics clinics was greater than 50 miles distance from center). Providers in these four health systems who had diagnosed patients with early onset female breast cancer (≤50 years of age), male breast cancer at any age, or ovarian cancer (including primary peritoneal and fallopian tube cancer) at any age were identified using data from the Michigan Cancer Surveillance Program (MCSP), the state’s cancer registry. Reporting all primary cancer diagnoses to the MCSP is required by state law [26]. Cancers in 80 Michigan counties were reported directly to the MCSP. Cancers in three counties were reported to the MCSP through the National Cancer Institute-funded SEER (Surveillance, Epidemiology and End Results) registry.

Using MCSP data on cases diagnosed in 2008–2009, the total number of cases appropriate for GC for HBOC in the state and at each of the four centers was determined. MCSP worked with the local cancer registrars of the four centers to identify the diagnosing providers for each of the cases. Provider-specific reports were generated by MDHHS to highlight the specific number of cases diagnosed by each provider with the total number of cases in the state. The profiles contained the number of cases that the provider had diagnosed from 2008–2009 that met the HBOC risk criteria. Patients’ names were not included in the report, but could be obtained by contacting the cancer registrar at each center.

Providers were mailed the provider-specific reports with a cover letter explaining the project and a 25-page booklet of HBOC educational materials and information about the new local cancer genetic clinic including contact information (collectively referred to as the provider packet). Suggestions for provider next steps once the provider packet was reviewed were also included. In the cover letter, providers were invited to contact their local genetic counselor to find out about the availability of services. They were also invited to contact the MDHHS education coordinator to discuss the report or to schedule a training session on cancer genetics.

Approximately six months after sending the provider packets, the providers’ offices were contacted up to three times by telephone to request follow up either by a telephone or an online survey. A copy of the provider packet was resent to interested providers’ offices. The follow-up survey contained 11 questions aimed at identifying the perceived value of the information sent and whether it impacted their knowledge or patterns of referral for patients at high risk for HBOC to genetic services. Additionally, the genetic counselors working at the newly established cancer genetic clinics were interviewed by telephone to determine whether they had received any feedback from providers and/or a change in the number of referrals in response to the implementation of this project. The interviews consisted of five questions and were conducted using a semi-structured format. A copy of the provider packet with a de-identified provider report was sent in advance for review. Interviews were electronically transcribed in order to summarize the qualitative responses.

### 2.2. Online Educational Module

In 2013, the MDHHS, in partnership with the CDC, Jackson Laboratory, Michigan State University, Oregon Health Department, Georgia Health Department and the Moffitt Cancer Center, developed an online educational module called “HBOC: Is Your Patient at Risk?” for primary care physicians and other non-genetics health care providers. An expert panel, comprised of public health professionals, a physician, genetic counselors, and educators took part in the development and review of the content [27]. The educational module focuses on four main areas of knowledge and skills: risk assessment, genetic testing, management, and collaboration. Each of these main topics is further divided into subtopic areas. The online module includes seven interactive cases, each with specific learning objectives. Each case includes a pedigree with additional case information, including links to useful tools available to help users navigate through the case. Users are asked to choose between three presented options to decide how they would manage the case based on the information provided. Each choice takes the user through a resulting outcome based on the answer chosen. If the answer chosen is incorrect, information is given as to why it is a wrong choice and the user is given the opportunity to choose another answer. When the correct answer is chosen, the reason it is correct is explained. 

The format of the educational module allows clinicians to access material on their own schedule, to access the material and tools most relevant to their practice (targeted education and point-of-care tools), and to proceed at their own pace. The module was approved for up to two American Medical Association Physician Recognition Award Category 1 continuing medical education credits (CME) provided by Michigan State University. The CME credits are available at no cost to users who complete a 10-question post-test and answer at least 7 out of 10 questions correctly.

Site analytic data for the first 12 months of the online module was analyzed using descriptive statistics. The data include the number of users who have accessed the educational module, which links were accessed, which cases, topics, and tools were viewed as well as materials downloaded. Data from users who applied for CME credit was collected including provider type, current practices with regard to risk assessment, referral and testing for HBOC, test scores, and overall satisfaction with the educational module. Non-identifiable data from CME users was analyzed using descriptive statistics.

## 3. Results and Discussion

### 3.1. Provider Bidirectional Reporting Results

Using MCSP data, in 2008–2009 there were 5005 cancers that were appropriate for GC based on HBOC referral guidelines. The breakdown of the number of early onset breast cancer cases, ovarian cancer cases and male breast cancer cases is shown in Table 1. The four centers targeted in this project reported 475 of these 5005 diagnoses (9.5%) (Table 1). Overall, the diagnoses at these four centers represented 11% of the early onset female breast cancers in Michigan in the 2008–2009, 7% of ovarian cancers, and 2.8% of male breast cancers.

A total of 69 providers were identified and mailed provider packets with individualized BDR reports in 2013. Of note, an additional 16 providers who had cases that met reporting criteria (16/85, 18.8% of total providers) did not receive packets because they were identified as being lost to follow up at the time the providers’ list was generated. Table 2 compares the areas of practice of diagnosing physicians by center and cancer type. Diagnoses were most often reported by general surgeons, hematology/oncology physicians, gynecologic oncologists or radiation oncologists. In many cases, a single provider or small number of providers reported a majority of the cases at a center. For instance, at Center 1, 97.7% (42/43) ovarian cancers were diagnosed by a single gynecologic oncologist and 47.1% (32/58) early onset breast cancers by a single general surgeon. At Center 4, 96.4% (27/28) of all early onset breast cancer and all ovarian cancer diagnoses were made by one of three hematology oncology physicians. But in other cases, diagnoses were split across a number of different providers/provider types. For instance at Centers 2 and 3, breast and ovarian cancer diagnoses were split amongst a variety of providers (Table 2).

Efforts to obtain feedback on the BDR initiative resulted in the project staff establishing contact with 24 different offices representing 35 of the 69 physicians who were sent provider-specific packets. Eleven of the 69 (15.9%) providers were lost to follow up for various reasons including a disconnected telephone number, retirement, left practice, medical leave or loss of medical records/no way to contact patients. An additional five providers (general surgeons) reported no longer performing breast surgeries and declined providing feedback. The remainder of offices did not respond after repeated attempts (3) to establish contact. In total, 19 offices representing 28 physicians agreed to have the survey sent either electronically, by fax, or by mail in order to potentially provide feedback. However, only five of these 28 practicing physicians provided feedback either by telephone or the online survey (7.2% of total number of physicians targeted). Their suggestions for improving the bidirectional reporting process included: timelier receipt of case data as the 4–5 year lag between diagnosis and reporting made acting on the information challenging; and more user-friendly formatting of the provider packet (too lengthy). However, given the low response rate, we cannot determine to what extent these comments are representative of those of all providers targeted.

Genetic counselor feedback was requested and received from each of the four genetic counselors at these centers. In general, the genetic counselors could not evaluate whether the bidirectional reporting project resulted in a change in the number of referrals. Three noted that referrals did increase during the period of time after the packets were sent; however, these increases could have been related to other efforts to increase appropriate referrals. Suggestions for ways to improve the bidirectional reporting included: sending the reports directly to the genetic counselors; reaching out specifically to those providers who see the most patients at risk; and notifying providers in advance of sending the packets that the information is coming to increase the likelihood they will review it once received.

### 3.2. Online Educational Module Results

In February 2014, the CME online module was launched and disseminated through multiple venues by MDHHS and their partners. From February 2014 through January 2015, 1835 unique users accessed the educational module over 2248 sessions. Users visited an average of 3.95 pages per session and the average time spent navigating the website was approximately 3.5 min. The website was most commonly accessed directly (through site URL) (55% (1241/2237) or through Google (30.5%, 683/2237).

Information regarding the institutions or internet service providers from which users came was available for 494 users. Users came from 47 different institutions located in 22 U.S. states and three countries outside the U.S. Institutions in the five states in which the module developers resided were over-represented (12/47).

The cases were reviewed 1369 times. The first case on the home page, that of “Marcus, the unsuspecting male”, was viewed most often and accounted for 22% of case views (300/1369). The least viewed case was “Geeta, a case of survivor’s guilt” (8% of case views; 110/1369). Geeta’s case is not on the home page and is the last case listed on the case studies page.

There were 2,724 views of the topic pages with the most commonly viewed related to risk assessment (46.5%, 1266/2724) (Table 3). The most used links in the educational module were those that informed the users about risk assessment, red flags that could indicate that a patient is at higher risk for developing HBOC related cancer, and genetic testing information. Fact sheets (point of care tools) were viewed a total of 1624 times and downloaded 764 times. The most commonly viewed and downloaded tools were the red flags checklist (433 views; 140 downloads), the HBOC fact sheet (276 views, 159 downloads) and the results interpretation table (202 views, 106 downloads). The least viewed links included genetic testing in children, adjusting a patient’s risk for developing an HBOC-related cancer, and ethical, legal, and social implications (ELSI).

As of January 2015, 59 individuals completed the 10-question post-test and requested continuing medical education (CME) credit. Those requesting CME credit were primarily female (51/58 reporting gender, 87.9%) and MD’s (21/59, 35.6%) or nurse practitioners (15/59, 25.4%). The post-test responders represented 16 different practice specialties; the most commonly represented were oncology (10/59, 16.9%), medical genetics (8/59, 13.6%), and obstetrics and gynecology (7/59, 11.9%). They practiced in suburban (25/54, 46.3%), urban (18/54, 33.3%) and rural (11/54, 20.4%) settings. A majority had 16 years or more experience (35/56, 62.5%).

A total of 43/59 (72.9%) of the CME participants passed the post-test with a score of seven out of 10 or better; the average score was 7.49. The two questions most likely to be answered correctly were the true/false questions on paternal transmission of a *BRCA* mutation (93.2%, 55/59) and the reduced penetrance of HBOC (94.9%, 56/59). The four questions most likely to be answered incorrectly were those pertaining to identifying patients at low-risk for HBOC (66.1%, 39/59 answered correctly), the types of cancers associated with HBOC (57.6%, 34/59 answered correctly), whether to test for a variant of unknown significance (VUS) (55.9%, 33/59 answered correctly), and the significance of a normal genetic test result in a person with a family history of a known *BRCA* mutation (67.8%, 40/59 answered correctly).

At the end of the quiz, the CME users were asked questions about their satisfaction with the online educational module. Using a five-point Likert scale, most respondents (94.9%, 56/59) indicated the case scenarios were a useful way to learn, that the tools provided were useful in completing the program (86.4%, 51/59), and that the tools would be useful in their practice (79.7%, 47/59). On a five-point Likert scale, most participants (93.2%, 55/59) indicated the content was relevant to their practice and that the online presentation of information was an effective way to learn (91.5%, 54/59).

In order to try to assess the impact of the educational module, CME participants were asked whether their participation would change their practice and if so, in what way. The responses were split with 31/59 (52.5%) indicating they would change how they practice. The most common ways noted included better attention to family history (nine respondents), more referrals/use of genetic counselors (six respondents), and better risk assessments (four respondents).

### 3.3. Discussion

The purpose of the two MDHHS provider education initiatives was to increase provider awareness of appropriate indications for referral to GC for HBOC. Currently, only a fraction of individuals at risk for HBOC in Michigan are being seen for GC and GT [25]. Identifying individuals with HBOC has significant implications with regard to medical management of patients and their at-risk relatives. Therefore, timely identification is imperative.

#### 3.3.1. Bidirectional Reporting Discussion

The effectiveness of the bidirectional reporting project could not be determined. The low response rate amongst diagnosing providers, despite multiple attempts to contact and resending of project materials, prevented any meaningful evaluation of the program. Based on the significant attrition of providers in the five years between the time when diagnoses were made and reported back, a major limitation of BDR is the time lag between diagnosis and reporting. In spite of the shortcomings of this initiative, collecting provider-specific data from the cancer registries helped the MDHHS identify which providers are diagnosing the majority of patients at risk of HBOC. At some centers, one or two providers are seeing a majority of cancer patients appropriate for GC and GT for HBOC. In these cases, targeting the specific providers, through a revised version of bidirectional reporting with a shorter time lag or through some other mechanism, could help ascertain a majority of patients at risk. In other centers, cases were dispersed amongst providers. This finding emphasizes the differences between various cancer facilities across the state of Michigan in terms of who is seeing patients at risk of HBOC and the need for tailored approaches to increasing provider awareness.

#### 3.3.2. Online Educational Module Discussion

In contrast to the provider-specific bidirectional reporting, the online educational CME module reached a number of providers across a variety of institutions, practice specialties, professions, and geographic locations. The topics most commonly viewed were those related to risk assessment and GT. These topic choices are consistent with the findings of a survey investigating internists’ GT attitudes and practices. In that study, respondents indicated that they needed more training on when to order genetic tests and how to interpret results [28]. Few users accessed the two subtopics under the collaboration topic heading-role of genetics specialists and effective referral. It may be that there was confusion about what “collaboration” meant. However, the two most commonly viewed case studies included referral/collaboration topics so some users may have been exposed to the content in that fashion. Klitzman *et al.* (2013) [28] found that only 53.4% of the internists they surveyed knew of a geneticist/genetic counselor to whom they could refer. Having a geneticist/counselor to whom to refer was associated with a significantly higher likelihood of ordering genetic testing [28]. A study of primary care physicians associated with a single insurance carrier found that among those who did not refer for genetic services, about 25% did not know how to initiate the referral process [20]. Strategies that enhance the proportion of views on collaboration topics may be beneficial to the overarching goal of increasing appropriate referrals.

About 73% of those taking the educational module quiz (post-test) for CME credit achieved a passing score, demonstrating adequate knowledge on the topics covered. Most post-test respondents answered questions about the importance of paternal family history and the reduced penetrance of HBOC correctly. Previous studies have shown that primary care physicians are often not aware of the importance of paternal family history in assessing risk for patients [21,29,30,31]. Whether the online educational module directly contributed to the knowledge the post-test respondents demonstrated on this topic cannot be definitively determined, though, since there was no pre-intervention assessment.

The questions most often answered incorrectly were those that concerned the interpretation and management of a variant of unknown significance (VUS), significance of a negative (normal) result in a family with a known BRCA mutation, identifying a low-risk patient, and identifying other cancers related to HBOC. With regard to variant interpretation and recognizing a “true negative” result, these are complex topics that likely require the development of additional provider education content and tools beyond what was provided in the online module. With regard to appropriately classifying a low risk history, which about one third of the post-test respondents were unable to do, previous studies have shown that primary care physicians frequently misclassify low risk family histories [19,22]. Discriminating between high and low risk patients is important because referring low risk individuals can actually be harmful (United States Preventive Services Task Force Grade D recommendation) [14,15]. Including additional low risk case scenarios in the online educational module may be necessary to help providers further develop this skill.

Four levels of evaluation for training programs have been previously described which include learner satisfaction (reaction-level 1), learning outcomes (learning-level 2), performance improvement (behavior-level 3) and patient/health outcomes (results-level 4) [32,33]. The evaluation measures available for the *HBOC:* Is your patient at risk? Module include satisfaction data, the CME test results (post-intervention knowledge only), and reported intent to change practice. Satisfaction was high in all the areas evaluated, including usefulness and relevance and most CME test takers indicated that the online format was an effective way to learn.

Although a majority of those seeking CME earned a passing score on the test, since there was no pre-education assessment, it is not possible to evaluate whether this outcome was a result of the online education or previous knowledge. There is also no way to determine at this time what factors may have influenced pass rate. Potential factors could include time spent reviewing online materials, previous experience in cancer genetics, professional degree, and/or practice area. Further analyses of demographic factors affecting pass rate may be beneficial once the number of CME test takers increases.

With regard to intent to change practice (enhance performance), about half of CME users indicated that reviewing the online module would change their practice in areas such as attention to family history, risk assessment and referral. The reasons that about half indicated they would not change practice are unknown. 

Overall, the evaluation of the educational module is limited by the fact that beyond web analytics, feedback was only obtained from those who requested CME credit. How the profile of the CME users compares to the full set of module users or the intended target audience—all providers with patients at risk of HBOC—is unknown. Requesting demographic information and providing an opportunity for feedback from all of those who view the educational module would be beneficial in getting a larger scale view of its potential reach and impact on awareness/referrals.

Despite the fact that only a small subset of users requested CME credit, a significant proportion of users viewed and/or downloaded fact sheets (point of care tools). This suggests that the online module may have served an important educational purpose beyond being a continuing medical education opportunity.

## 4. Conclusions

We report on two different public health initiatives aimed at increasing provider awareness and identification of HBOC. The targeted approach of utilizing cancer registry data to providers (bidirectional reporting) was a lengthy and time-consuming process with uncertain impact. Nonetheless, the registry data provided important information about which providers are diagnosing the majority of cancer patients at risk for HBOC and how this varies amongst facilities. The broad-based interactive CME educational module reached over 1,800 unique individuals in one year’s time. Based on the subset of users seeking CME credit, a majority were able to demonstrate an adequate grasp of the material presented as evidenced by a passing score on the CME quiz. In addition, a small majority said taking the module would change how they practice. However, we cannot assess whether the module is being utilized by our target audience—providers with limited awareness of HBOC who see at risk patients. One way to reach these providers might be to use the cancer registry data to identify those diagnosing large numbers of cancer patients at risk for HBOC and reach out to them directly (e.g., in office visits) and repeatedly with information about the online CME module and other educational resources.

## Figures and Tables

**Table 1:** Number of cases at risk for HBOC per center, 2008–2009.

Cancer Center	Number of Providers at Center Receiving Reports	Cancer Cases seen in 2008–2009			
		Number of Early Onset Female Breast Cancers (Diagnosed ≤ 50)	Number of * Ovarian Cancer Cases	Number of Male Breast Cancer Cases	Total Cases
Center 1	18	68	43	1	112
Center 2	35	216	52	3	271
Center 3	12	47	17	0	64
Center 4	4	22	6	0	28
Total	69	353	118	4	475
State of MI Total		3184	1680	141	5005

* includes ovarian, fallopian tube, and primary peritoneal cancers.

**Table 2:** Number of cases per diagnosing provider type per center, 2008–2009 ^∫^.

Provider Type	Center 1		Center 2		Center 3		Center 4	
	Number of Early Onset Female Breast Cancers ≤ 50 (%) *	Number of Ovarian Cancers (%) *	Number of Early Onset Female Breast Cancers ≤ 50 (%) *	Number of Ovarian Cancers (%)	Number of Early Onset Female Breast Cancers ≤ 50 (%) *	Number of Ovarian Cancers (%) *	Number of Early Onset Female Breast Cancers ≤ 50 (%) *	Number of Ovarian Cancers (%) *
General Surgeons	62 (91.2%)	0	107 (49.5%)	1 (1.9%)	7 (14.9%)	0	0	0
Gynecologic Oncology	0	42 (97.6%)	1 (0.5%)	22 (42.3%)	0	2 (11.8%)	0	0
Hematology Oncology	1 (1.5%)	1 (2.3%)	95 (44.0%)	25 (48.0%)	26 (55.3%)	8 (47.1%)	21 (95.6%)	6 (100%)
Radiation Oncology	0	0	11 (5.1%)	0	14 (29.8%)	0	1 (4.5%)	0
Family Medicine	5 (7.3%)	0	1 (0.5%)	1 (1.9%)	0	1 (5.9%)	0	0
Obstetrics and Gynecology	0	0	0	2 (3.8%)	0	5 (29.4%)	0	0
Other	0	0	1 (0.5%)	1 (1.9%)	0	1 (5.9%)	0	0

* Percentages indicate the percent of all of the diagnoses of that type made by the specified provider type at that Center. ^∫^ Male breast cancer diagnoses are not included in this table given the small number of diagnoses.

**Table 3:** Number of webpage views by main topics and subtopics *.

Main Topics	Subtopics (in Bold) and Specific Subject Areas (in Italics)	# of Page Views Total = 2724
Risk Assessment		312
	Collect Risk Information Includes targeted genetic interview, risk assessment challenges, cancers associated with HBOC, and other hereditary cancers	434
	Identify Red Flags	194
	Assess Risk Includes HBOC inheritance pattern, paternal family history, stratifying risk, adjusting risk	326
*Total views related to risk assessment topics = 1266 (46.5% of all topic reviews)*		
Genetic Testing		160
	Testing Strategy Includes testing affected relative first, ancestry matters, test methods	336
	Interpretation Includes interpreting a positive result, a negative result, and a VUS	313
	Considerations Includes ELSI implications and testing in children	129
*Total views related to genetic testing topics = 938 (34.4% of all views)*		
Management		117
	Screening Guidelines	100
	Risk Reduction Strategy	110
	Testing & Screening Relatives	52
*Total views related to management topics = 379 (13.9% of all views)*		
Collaboration		0
	Role of Genetic Specialists	65
	Effective Referral	76
*Total views on collaboration topic = 141 (5.2% of all views)*		

* Of note, those visiting the topics could link directly to the main topic (e.g., risk assessment), the subtopic (e.g., collect risk information), or the specific subject areas.
